# *LRP1B* or *TP53* mutations are associated with higher tumor mutational burden and worse survival in hepatocellular carcinoma

**DOI:** 10.7150/jca.48983

**Published:** 2021-01-01

**Authors:** Longrong Wang, Kai Yan, Xigan He, Hongxu Zhu, Jia Song, Shiqing Chen, Shangli Cai, Yiming Zhao, Lu Wang

**Affiliations:** 1 Liver Surgery Department, Shanghai Cancer Center, Fudan University; Department of Oncology, Shanghai Medical College, Fudan University, Shanghai 200032, China.; 2 Fifth Department of Liver Surgery, Shanghai Eastern Hepatobiliary Surgery Hospital, Second Military Medical University, Shanghai, China.; 3 The Medical Department, 3D Medicines Inc., Shanghai, China.

**Keywords:** Hepatocellular carcinoma, *LRP1B*, * TP53*, tumor mutational burden, survival

## Abstract

**Background:** Hepatocellular carcinoma (HCC) is one of the most leading causes of cancer-related mortality worldwide. Immune checkpoint inhibitors (ICIs) have been proved to be beneficial for advanced HCC. Tumor mutational burden (TMB) is an important predictor for efficacy of ICIs. However, the genetic landscape of Chinese HCC patients and the association between TMB and frequently mutated genes of HCC remain unclear.

**Methods:** Whole-exome sequencing data of 369 liver tumors from the Cancer Genome Altas (TCGA) and next generation sequencing (NGS) data of 657 liver tumors from Chinese clinical dataset were included.

**Results:** *TP53* (61.8%) was the most frequently mutated gene in the Chinese cohort, followed by *CTNNB1* (17.2%), *RB1* (13.7%), and *LRP1B* (12.3%). The PI3K-Akt signaling (11.2%), the Rap1 signaling (8.1%), and Ras signaling (7.7%), were significantly mapped. *LRP1B* mutations were significantly associated with higher TMB in both TCGA cohort (*P* = 0.0003) and Chinese cohort (*P* = 0.0005). And *TP53* mutations were also associated with higher TMB in the TCGA and Chinese cohort (*P* = 0.0005 and 0.0010, respectively). Prognosis analysis performed in TCGA cohort revealed *LRP1B* mutations were significantly associated with shorter overall survival (OS, median, 20.9 vs 61.7 months; HR, 2.22; *P* = 0.0012). *TP53* mutation was an independent risk factor affecting both OS (HR 1.58,* P* = 0.0109) and PFS (HR 1.59, *P* = 0.0027).

**Conclusions:** The results suggest that *LRP1B* or *TP53* mutations are associated with higher TMB and a poor prognostic factor in HCC.

## Introduction

Hepatocellular carcinoma (HCC) is a heterogeneous disease with rising incidence and mortality rate 1. Surgery and liver transplantation are the potentially curative treatments; however, only less than 40% of the patients are diagnosed at the early stage 2. For patients with advanced stage, systemic therapies are the cornerstone. The multi-target tyrosine kinase inhibitor (TKI) Sorafenib was the unique systemic regimen which had been approved between 2007 and 2016 3. Recently, several promising drugs have showed clinical activity in patients with HCC, like levatinib in the frontline 4 and ramucirumab 5, regorafenib 6 and cabozantinib 7. However, the objective response rates (ORRs) were no more than 15%, which is still not enough to meet the clinical requirements. Immune checkpoint inhibitors (ICIs) have demonstrated clinical benefit in multiple solid tumors, which have been currently approved by FDA 8, 9. What's more, several researches have proven that patients with high tumor mutational burden (TMB) generally presented better response to ICIs than the rest 10-12.

TMB is a measurement of the number of mutations harbored by tumor cells, and is usually determined by using next-generation sequencing (NGS). Several studies have revealed that alternations in genomic integrity related genes might result in genomic instability and replication stress 13, 14. The transcription factor TP53, encoding p53 protein, activates in response to multiple stressors and regulate the expression of genes controlling proliferation, DNA repair, and cell death 15. As a major tumor suppressor, *TP53* is highly prevalent on genic alternations in diverse cancer types 16. The low-density lipoprotein receptor-related protein 1B (LRP1B), encoding endocytic low density lipoprotein family receptor, commonly binds to extracellular ligands and is characterized as a candidate tumor suppressor 17.

Currently, the characteristic mutational landscape of HCC has been revealed by several researches 18-20. However, the association between the genomic feature and TMB or prognosis have not been deeply probed. Herein, we showed the genomic features in Chinese HCC patients based on NGS, analyzed the effect of gene mutations on TMB in a Chinese HCC dataset and the TCGA dataset. In addition, we further revealed the prognosis of patients harboring *LRP1B* or *TP53* mutations in TCGA.

## Materials and Methods

### Sample collection and clinicopathologic data

From January 2017 to November 2018, tumor specimens and matched blood samples from 657 patients with primarily diagnosed as HCC were obtained in Fudan University Shanghai Cancer Center. Genomic profiling was tested in a CAP-certified/CLIA-accredited laboratory (3D Medicines Inc., Shanghai, China). Clinicopathologic information, including sex and age were collected. The present study was approved by the Ethics Committee of the hospital, and a waiver of informed consent form was signed by all the patients.

### DNA extractions, targeted sequencing and data processing

The assay methodology of DNA extraction and sequencing was followed the methods published in previous paper with some modifications 21. Namely, tumor genomic DNA was extracted using QIAamp DNA FFPE Tissue kit (Qiagen GmbH, Hilden, Germany), and normal genomic DNA was extracted from peripheral blood mononuclear cells using QIAamp DNA Blood Mini kit (Qiagen GmbH, Hilden, Germany), respectively, following the manufacturer's protocols. Next-generation sequencing (NGS) targeted 381 cancer-related genes were performed on the NextSeq500 platform (Illumina, CA, USA), and samples with a mean coverage depth after de-duplicating reads of 500× was analyzed. Using BWA aligner (version 0.7.12), sequencing data were mapped to the human genome 19 (hg19). After that, the data experienced the process of PCR duplicate read removal and sequence metric collection with the aid of Picard (version 1.130) and Samtools (version 0.1.19). The clean data were analyzed to identify diverse genic alternations, like base substitution, indel, rearrangement, and copy number variant, by analysis pipelines developed by 3D Medicines Inc (Shanghai, China). Tumor mutational burden (TMB) was defined as total number of somatic non-synonymous mutations in coding region. Data from TCGA from cBioPortal (https://www.cbioportal.org/) was extracted in February 2019.

### Statistical analysis

The demographic characteristics of patients were compared via the Chi-Square (χ^2^) test or T test. All *P*-values presented were two-sided, and associations were considered significant if the *P*-value was less than 0.05. Functional enrichment analysis, including Gene Oncology (GO) and signaling pathway, were performed by T test. Gene Oncology (GO) and pathway analysis on gene alternations were performed using DAVID (https://david.ncifcrf.gov/), and drawn in R by using the package “ggplot”. Overall survival (OS) and progression-free survival (PFS) were analyzed using the Kaplan-Meier method with a *P* value determined by the log-rank test, and drawn with GraphPad Prism version 6 (GraphPad Software Inc., LA, CA, USA). Hazard ratios (HR) were estimated using Cox proportional hazards regression. All statistical analyses were performed using the SPSS statistical package, version 20.0 (SPSS Inc^®^, Chicago, Illinois, USA).

## Results

### Patient characteristics

A total of 657 Chinese patients with HCC and 372 HCC patients from the TCGA database were included in this study. The basic characteristics were shown in **Table 1**, and not significant difference on sex and age existed between these two datasets. The median age was 53 years (range, 16-82) in the Chinese dataset and 61 years (range, 16-90) in the TCGA dataset. The percentage of male was respectively 87.2% and 68% in the Chinese dataset and TCGA dataset.

### Detection of gene alternations

**Figure 1:**
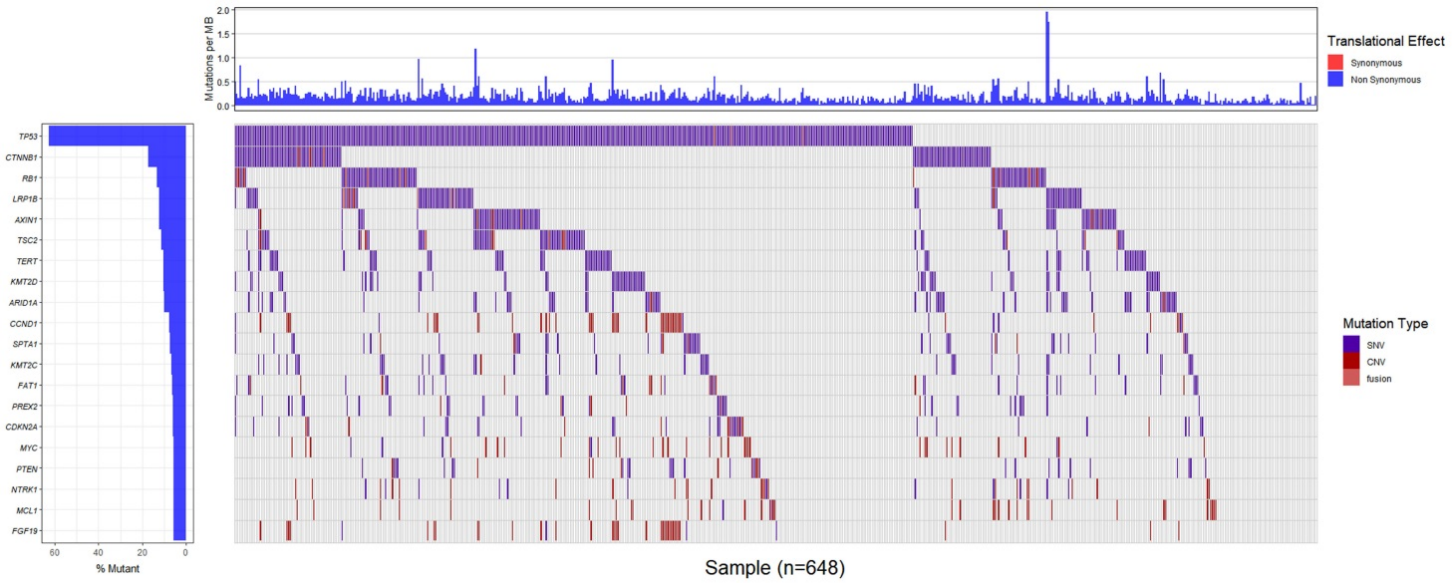
Genomic alternations of the Chinese patients with hepatocellular carcinoma.

**Figure 2:**
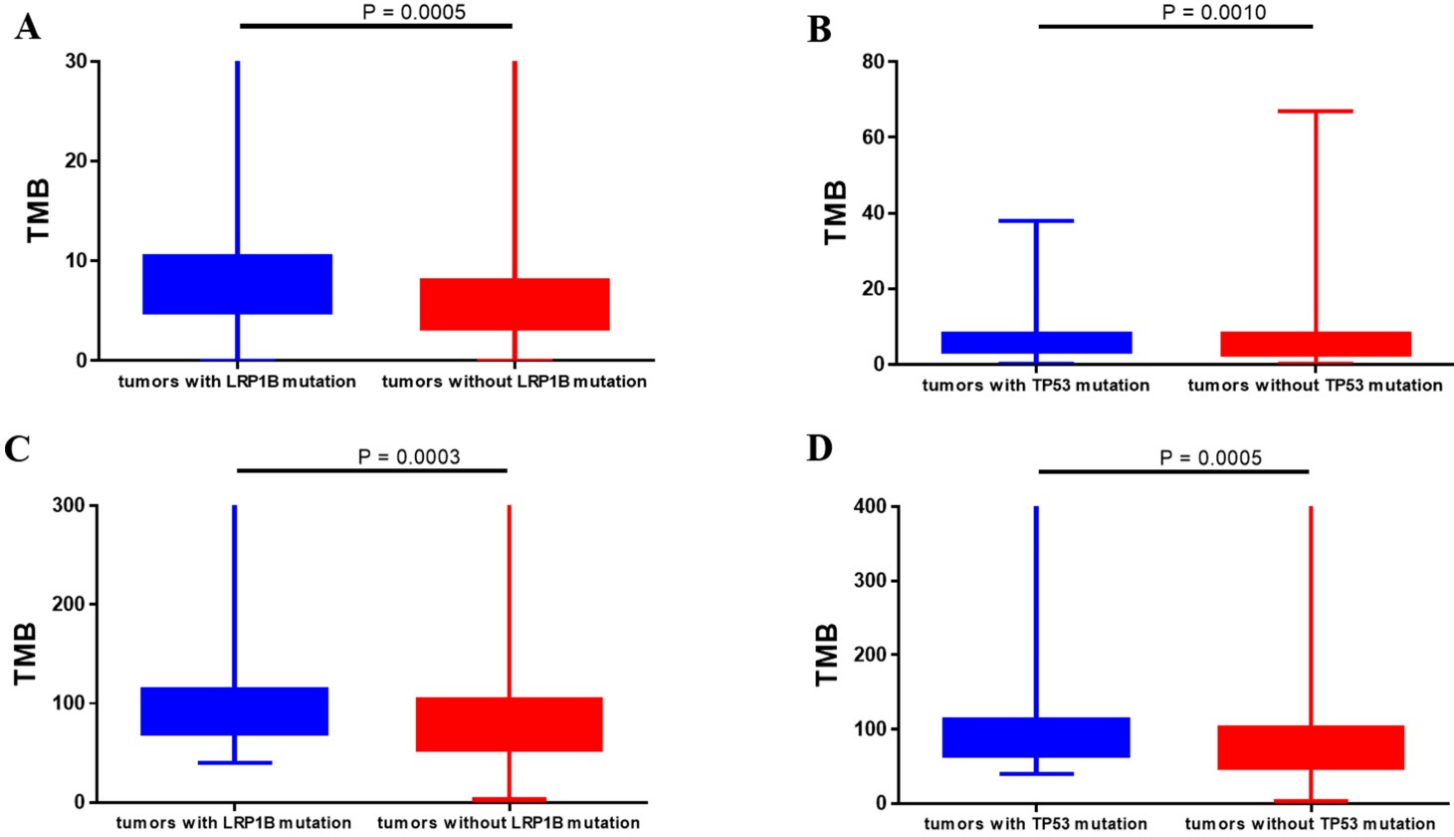
Correlation between gene alternations and tumor mutational burden. (A, B) Chinese clinical cohort. (C, D) TCGA dataset.

Totally, 648 patients (98.6%) in Chinese dataset detected somatic gene alternations. Significantly somatic genic alternations in tumor samples were identified and the genomic profile was drawn in **Figure 1**. *TP53* (61.8%) was the most frequently mutated gene, followed by *CTNNB1* (17.2%), *RB1* (13.7%), and *LRP1B* (12.3%). Patients harboring *LRP1B* mutations presented significantly higher TMB than that with *LRP1B* wild-type (*P*=0.0005), and the same result was also observed in patients with *TP53* mutations (*P*=0.0010, Figure 2A and 2B). What's more, the parallel results were also observed in the TCGA dataset. The frequency of *TP53* mutations and *LRP1B* mutations in TCGA cohort was 28.7% (106/369) and 9.5% (35/369), respectively. The *LRP1B* and *TP53* mutations were significantly associated with a higher TMB (*P*=0.0003 and 0.0005, respectively, **Figure 2C** and **2D**).

### GO and pathway analysis

**Figure 3:**
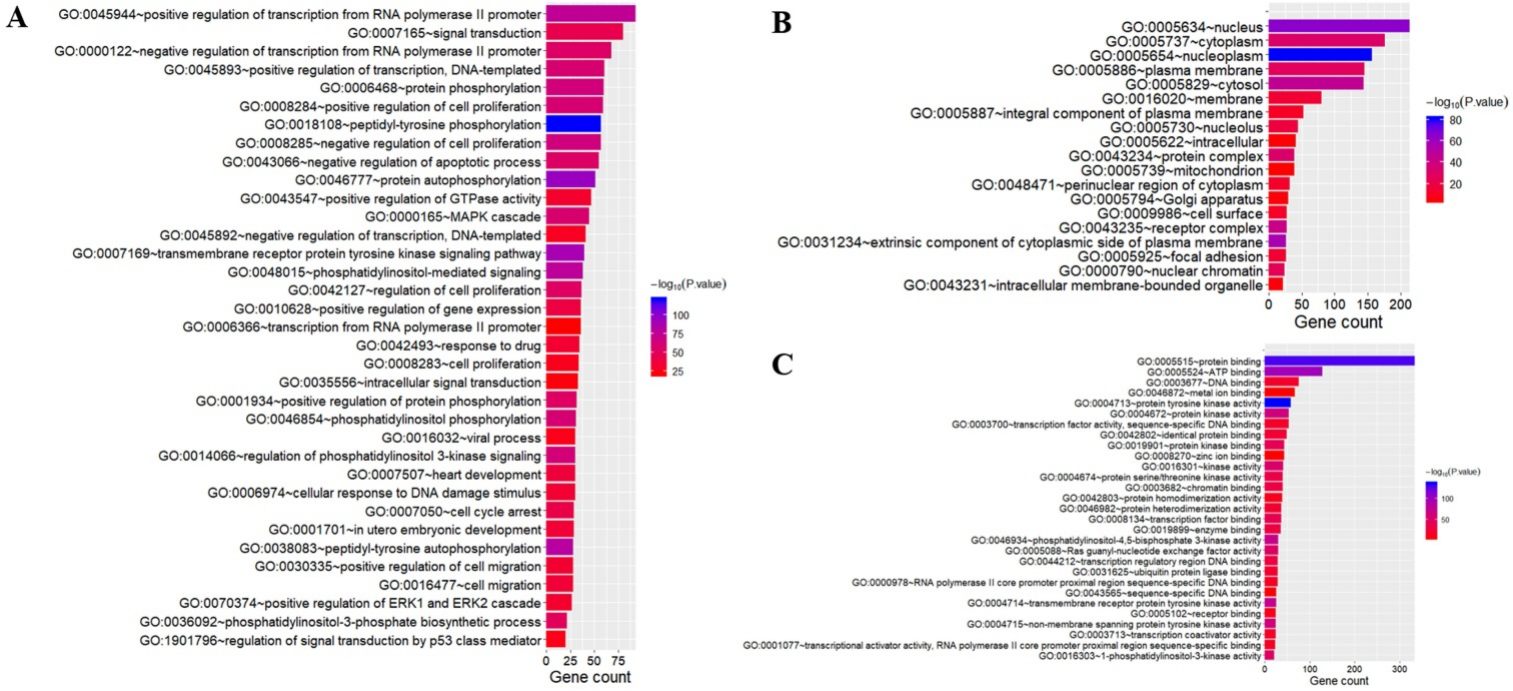
Gene oncology analysis of highly altered genes. The terms of biological process (A); the terms of cellular component (B); the terms of molecular function (C).

**Figure 4:**
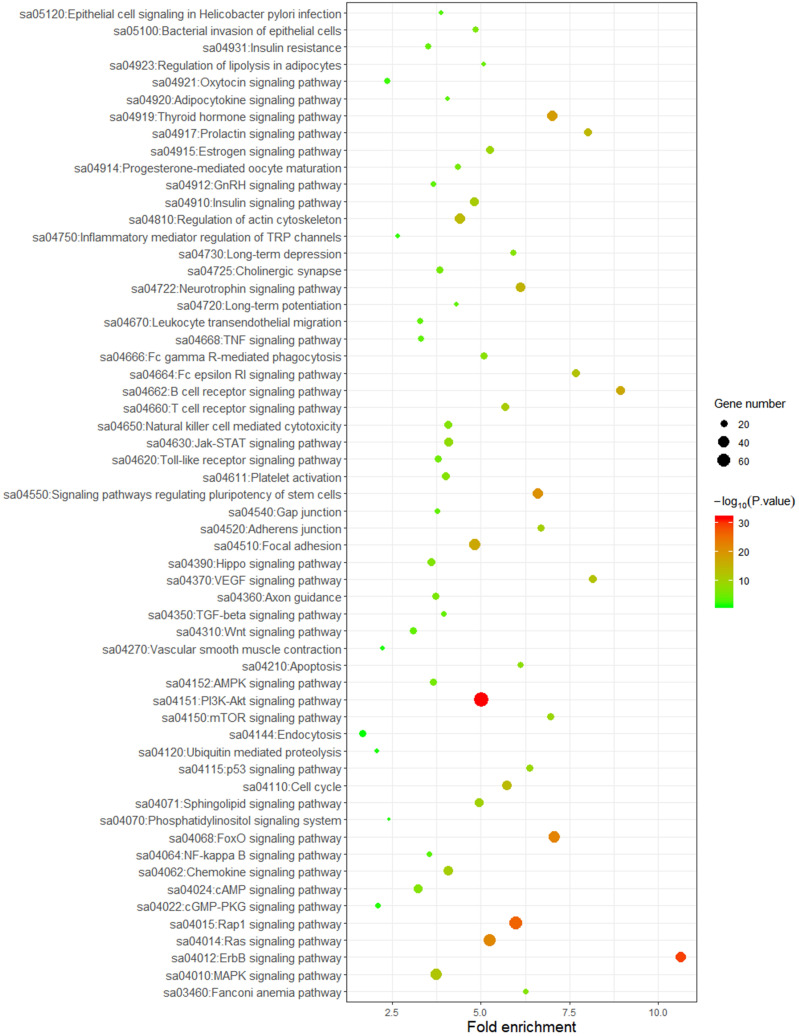
Mapping pathways by frequently mutated genes.

To better understand the biological function of these highly frequent somatic alternations in HCC, GO enrichment and signaling pathways analysis were performed. The significantly enriched GO terms of biological process associated with regulation of transcription, regulation of cell cycle, and the process of cellular signal transduction. Most of the genes located on nucleus and cytoplasm, and the top mapped molecular functions were protein binding, ATP binding, DNA binding, and metal ion binding (**Figure 3**). As shown in **Figure 4**, the pathways that may be implicated in HCC were commonly mapped. The PI3K-Akt signaling (11.2%) enriched the most altered genes, followed by the Rap1 signaling (8.1%), and Ras signaling (7.7%). In addition, several pathways related with cellular process, like cell cycle, apoptosis, immune microenvironment associated with pathways, and B cell/T cell receptor signaling pathways, were also significantly mapped. Although *LRP1B* and *TP53* significantly impacted the TMB value, no obvious difference on signaling pathways existed between the patients with mutation and wild-type.

### *LRP1B* or *TP53* mutations predicted worse prognosis

**Figure 5:**
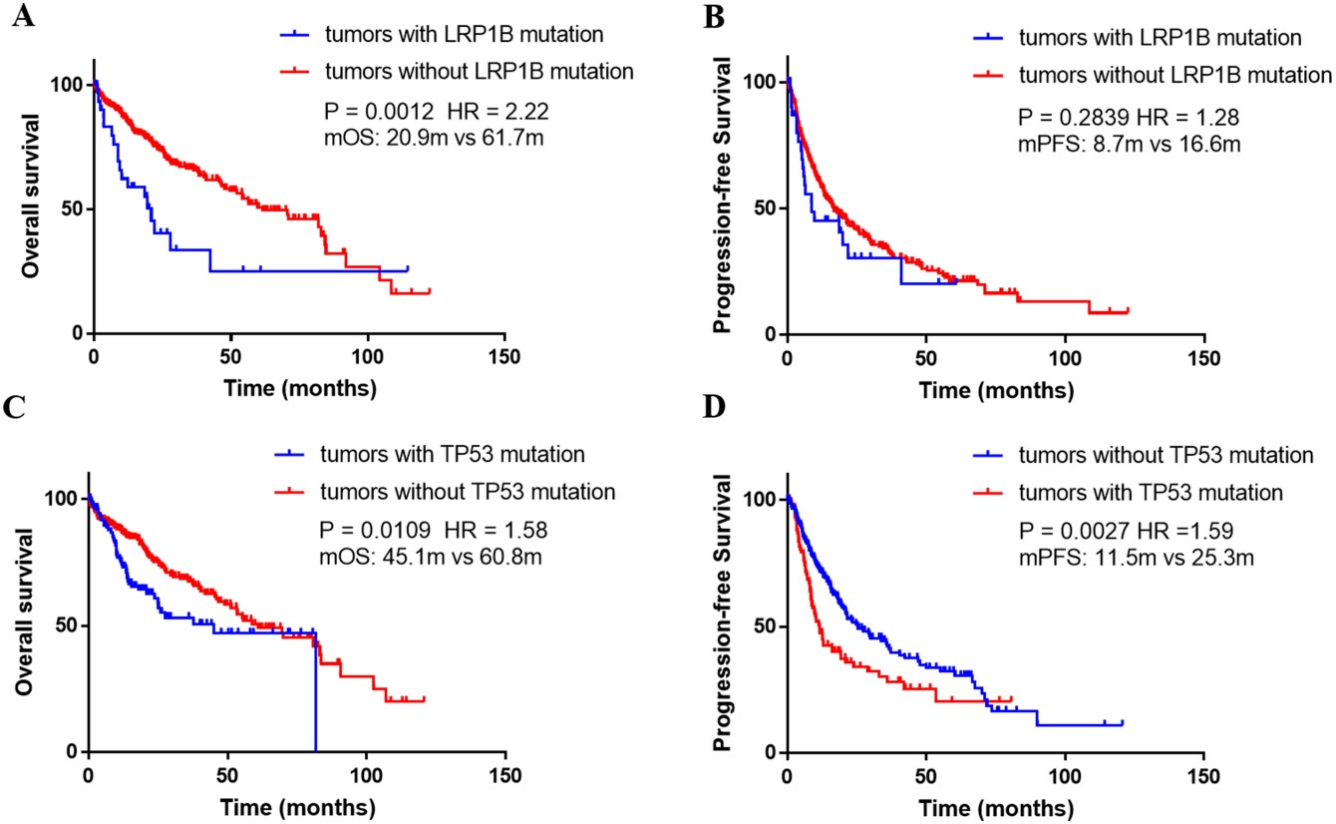
Association between gene alternations and survival outcomes in the TCGA dataset**.** (A) Kaplan-Meier survival curves of overall survival comparing patients with wild-type* LRP1B* and patients with mutated *LRP1B*. (B) Kaplan-Meier survival curves of progression-free survival comparing patients with wild-type* LRP1B* and patients with mutated *LRP1B*. (C) Kaplan-Meier survival curves of overall survival comparing patients with wild-type *TP53* and patients with mutated *TP53*. (D) Kaplan-Meier survival curves of progression-free survival comparing patients with wild-type *TP53* and patients with mutated *TP53.*

Using the Kaplan-Meier curve analysis, in the TCGA dataset, the overall survival (OS) was significantly shorter in the patients harboring *LRP1B* mutations than that with wild-type (median, 20.9m versus 61.7m, *P*=0.0012, HR=2.22, **Figure 5A**). In addition, median PFS was numerically shorter in the patients with *LRP1B* mutations than the rest of the patients (median, 8.7m versus 16.6m, *P*=0.2839, HR=1.28, **Figure 5B**). What's more, *TP53* mutated patients presented significantly shorter OS and PFS compared with wild-type patients (median OS, *TP53*-mut versus *TP53* wild-type: 45.1m vs 60.8m, *P*=0.0109, HR=1.58; median PFS, *TP53*-mut versus *TP53* wild-type: 11.5 m vs 25.3 m, *P*=0.0027, HR=1.59, **Figure 5C** and **5D**).

## Discussion

The present study revealed the genomic alternations of Chinese patients with HCC, the possibly biological function of altered genes, the association between the TMB value and *LRP1B*/*TP53* mutations, and the impact of *LRP1B*/*TP53* mutations on prognosis. These results indicate that *LRP1B*/*TP53* mutations might be the prognostic predictors and associated with higher TMB, predicting better efficacy of immunotherapy in HCC patients.In our study, *TP53* and* CTNNB1* were the most frequently mutated genes, followed by *RB1* and *LRP1B*. This result was similar with previous studies. A Whole-genome sequencing research indicated that *TP53*, *CTNNB1*, and *RB1* were most common mutated among the protein-encoding genes, and frequently mutations were also observed in *LRP1B* which could be used as one of the mutational signatures to classify molecular subtypes in Japanese patients with liver cancer 18. Another study also showed the tumor suppressor genes, including *TP53* and* RB1*, and the oncogenes, like *CTNNB1*, were the significantly mutated genes in HCC patients 19. Certainly, a relatively higher frequency of *TP53*/*RB1* alternations were observed in our Chinese dataset, which is consistent with another research which included a large number of the Chinese HCC patients 22. For the mapped pathways of our work, besides the reported PI3K-AKT signaling and Ras signaling pathways 19, 23, we also reported the Rap1 signaling which plays an important role in tumor processes, such as cell migration, invasion, and metastasis 24.

We identified that patients harboring *LRP1B* and *TP53* alternations presented higher TMB, respectively. This two genes were both frequently mutated in multiple types of human cancer. Chen and his colleagues have revealed that higher TMB was found in* LRP1B* mutated patients with melanoma and non-small cell lung cancer 25. An integrated analysis on the multiple-dimensional data types indicated that significantly increased non-synonymous mutations generally presented in the *TP53-*mutated group compared with the wild-type group 26. Although several researches have probed the relationship between *LRP1B*/*TP53* and TMB, no solid conclusions existed on HCC. In addition, based on the public database, the *LRP1B* alternations correlating with poor survival was also revealed. In patients with glioblastoma, *LRP1B* is generally down expressed, and *LRP1B* deletion is associated with poor outcome 27. However, to our knowledge, no definite conclusion on the correlation between *LRP1B* and prognosis in HCC has been made, and certainly, our results are needed to be validated further.

One limitation of this paper is failure to collect the whole baseline clinical characteristic and therapeutic regimens. And another limitation was no prognosis data from the Chinese clinical dataset. Thus, the further research need to focus on the association between gene alterations and clinical outcomes in Chinese HCC cohort.

To sum up, our work revealed the genomic landscape of HCC with a large sample size, probed the association between gene alternations and TMB, and pointed out the impact of specific gene alternations on prognosis. These results might aid to screen potential therapeutic targets and better manage the whole disease process.

## Figures and Tables

**Table 1:** Characteristics of hepatocellular carcinoma patients from the clinical dataset and the TCGA dataset

Characteristic	Clinical dataset	TCGA dataset
Cases	657	369
Median age, year (range)	53 (16-82)	61 (16-90)
Sex (male vs female)	573 vs 84	251 vs 118
*LRP1B* (mutation vs wild-type)	81 vs 576	35 vs 334
*TP53* (mutation vs wild-type)	406 vs 251	106 vs 263
